# Question of Sex Education

**Published:** 1912-10

**Authors:** 


### Question of Sex Education.

Apropos of Dr. Eby's classical paper in the last month's edition of the JOURNAL, "Should Sex Hygiene Be Taught in the Home and School?" we would quote the conclusion arrived at by the joint commission of the National Education Association and the Federation of Women's Clubs. This report advises:

"It is generally conceded by educators, physicians and social workers that there is urgent need for instruction in personal and sex hygiene in the schools.

"That such instructions should be grounded in biology.

"That it should include more than physical facts.

"That it is dangerous to introduce the subject generally into elementary and secondary schools until it is required in normal schools and teachers carefully selected and prepared for the work.

"That the normal schools are ready and willing to give such instruction as soon as educators can agree as to what are 'wise methods' and what are the 'essentials.'

"That instruction in personal and sex hygiene should be extended to parents and gradually to the general public.

"That popular prejudice against such instruction in the schools is rapidly disappearing.

"That such instruction is essential in eradicating the 'social evil' and controlling venereal diseases."

It would seem that it is no longer a question of propriety or expediency in instructing both young and old in sex hygiene, and that this instruction should be given in the public schools through teachers properly trained in the normal schools. The question of more difficult solution is in what way this education should be conducted. The American Federation of Sex Hygiene has set for itself the task of formulating a systematic course of study adapted to all classes and conditions. To this end the co-operation of experts in physiology, psychology, biology, sociology, medicine and pedagogy have been solicited.

At the present time there are 138 schools in forty States that teach sex hygiene in some manner or other. One-third of the schools make the subject obligatory, while the rest leave the matter as an elective course. Most of the schools require separate classes. Everywhere, through the influence of mothers' clubs, social hygiene societies, magazines and educational institutions, these questions are being agitated and placed fairly before the people.

